# Mapping Variation in Cellular and Transcriptional Response to 1,25-Dihydroxyvitamin D3 in Peripheral Blood Mononuclear Cells

**DOI:** 10.1371/journal.pone.0159779

**Published:** 2016-07-25

**Authors:** Silvia N. Kariuki, Joseph C. Maranville, Shaneen S. Baxter, Choongwon Jeong, Shigeki Nakagome, Cara L. Hrusch, David B. Witonsky, Anne I. Sperling, Anna Di Rienzo

**Affiliations:** 1 Department of Human Genetics, University of Chicago, Chicago, Illinois, United States of America; 2 Department of Medicine, University of Chicago, Chicago, Illinois, United States of America; College of Tropical Agriculture and Human Resources, University of Hawaii, UNITED STATES

## Abstract

The active hormonal form of vitamin D, 1,25-dihydroxyvitamin D (1,25D) is an important modulator of the immune system, inhibiting cellular proliferation and regulating transcription of immune response genes. In order to characterize the genetic basis of variation in the immunomodulatory effects of 1,25D, we mapped quantitative traits of 1,25D response at both the cellular and the transcriptional level. We carried out a genome-wide association scan of percent inhibition of cell proliferation (I_max_) induced by 1,25D treatment of peripheral blood mononuclear cells from 88 healthy African-American individuals. Two genome-wide significant variants were identified: rs1893662 in a gene desert on chromosome 18 (p = 2.32 x 10^−8^) and rs6451692 on chromosome 5 (p = 2.55 x 10^−8^), which may influence the anti-proliferative activity of 1,25D by regulating the expression of nearby genes such as the chemokine gene, *CCL28*, and the translation initiation gene, *PAIP1*. We also identified 8 expression quantitative trait loci at a FDR<0.10 for transcriptional response to 1,25D treatment, which include the transcriptional regulator ets variant 3-like (*ETV3L*) and EH-domain containing 4 (*EHD4*). In addition, we identified response eQTLs in vitamin D receptor binding sites near genes differentially expressed in response to 1,25D, such as FERM Domain Containing 6 (*FRMD6*), which plays a critical role in regulating both cell proliferation and apoptosis. Combining information from the GWAS of I_max_ and the response eQTL mapping enabled identification of putative I_max_-associated candidate genes such as *PAIP1* and the transcriptional repressor gene *ZNF649*. Overall, the variants identified in this study are strong candidates for immune traits and diseases linked to vitamin D, such as multiple sclerosis.

## Introduction

Epidemiological studies have linked variation in the circulating inactive form of vitamin D, 25-hydroxyvitamin D_3_ (25D), to risk of autoimmune diseases such as multiple sclerosis, type 1 diabetes and systemic lupus erythematosus [1–7], consistent with the known effects of vitamin D as an immune system modulator [8–12]. Furthermore, genetic variation in the vitamin D pathway is linked to autoimmune disease risk. For example, several studies have highlighted associations between variants in *CYP27B1*, which encodes the enzyme that activates 25D to 1,25-dihydroxyvitamin D_3_ (1,25D), and risk for multiple sclerosis [13–15].

The fact that immune cells express CYP27B1 indicates that active vitamin D can be produced intra-cellularly in the immune system in response to organismal demands such as infections. Immune cells also express the vitamin D receptor (VDR), which when bound by the active 1,25D, forms a heterodimer with the retinoid X receptor (RXR) and translocates to the nucleus, resulting in transcriptional regulation of vitamin D-responsive genes [8, 9, 11, 12, 16]. The genes regulated by 1,25D are involved in various pathways including metabolic regulation, antimicrobial response and inflammatory cytokine response [7, 17–23].

Extensive inter-individual and inter-ethnic variation in the circulating levels of 25D levels has been reported, with lower levels on average in African Americans compared to European Americans [24–27]. These differences are known to be influenced by various factors such as sun exposure, dietary intake, as well as genetic variations in critical genes in the vitamin D metabolic pathway [14, 28, 29]. Despite the strong epidemiological associations of 25D levels and disease risk, randomized clinical trials aimed at testing the efficacy of vitamin D supplementation as a therapeutic intervention [4, 17, 30–34] have yielded mixed results [35, 36]. In addition to environmental confounders, these results could be due to inter-individual differences in the response to vitamin D, irrespective of its concentration in circulation or within the cells at the level of the target organ. Indeed, at least one study identified a polymorphism in the *VDR* gene that influenced the response to vitamin D supplementation [37]. However, beyond the *VDR* gene, little–if anything–is known about the contribution of genetics to the inter-individual variation in response to vitamin D.

The aim of this study was to map the genetic bases of inter-individual variation in the transcriptional response to 1,25D and in the inhibition of cell proliferation induced by 1,25D in primary peripheral blood mononuclear cells (PBMCs) obtained from African-American healthy individuals. We primarily focused on African-American individuals as epidemiological data indicate that they have a higher proportion of 25D deficiency, and should hence be considered prime targets of supplementation studies, which in turn could benefit from knowledge of genetic variation affecting response to supplementation. Moreover, measuring the proportion of genome-wide African ancestry enabled us to test the relationship between African ancestry proportions and response to 1,25D within the same ethnic group. To isolate the effects of genetic variation on the response to active vitamin D rather than on its concentration, we treated PBMCs cultured *in vitro* with a fixed amount of 1,25D and, in parallel, with a vehicle control. This allowed us to characterize the response to vitamin D both at the cellular and transcriptional level and to identify genetic variants associated with cellular and transcriptional response to 1,25D. Moreover, by measuring the proportion of African ancestry in the African-American cohort in this study, we were able to directly test the relationship between African ancestry and response to 1,25D.

## Methods

### Samples

Peripheral blood was obtained from 88 African American (AA) donors collected by Research Blood Components (http://researchbloodcomponents.com/) as part of a larger study on transcriptional response [38]. All subjects were healthy donors and were not on any medication. All donors to Research Blood Components are required to sign an Institutional Review Board (IRB)-approved consent form giving permission to collect blood, and use it for research purposes. The IRB at the University of Chicago determined that this study is not human subjects research because blood samples were not shipped with individually identifiable information. Self-reported ethnicity, age, gender, date, and time of blood drawing were recorded for each donor (**S1 Table**). Samples were processed in multiple successive batches. Batch number was recorded and used as a covariate.

### Cell Culture and Treatment

The experimental design is illustrated in **S1 Fig**. We isolated peripheral blood mononuclear cells (PBMCs) from heparin-treated whole blood by density gradient centrifugation using Ficoll-Paque PLUS medium (GE Healthcare Life Sciences, Pittsburgh, PA), within 24 hours of blood draw for all the samples. PBMCs were washed in PBS and transferred to RPMI supplemented with 10% charcoal-stripped fetal bovine serum. Each sample was then divided into one aliquot of 1.8 x 10^6^ cells for measuring cell proliferation, and one aliquot of 9 x 10^6^ cells for genome-wide transcriptional profiling. For the cell proliferation measurements, PBMCs were cultured at 2 x 10^5^ cells per well in 10% charcoal-stripped media in 96-well plates. Each donor was treated in triplicate with phytohemagglutinin (PHA) (2.5ug/ml) and either vehicle (EtOH) or 1,25-dihydroxyvitamin D3 (1,25D) (100nM) for 48 hours [38, 39]. For transcriptional profile measurements, PBMCs from each donor were cultured at 10^6^ cells per well in 10% charcoal-stripped media in 24-well plates. As with the cellular proliferation measurements, each donor was treated in triplicate with PHA (2.5ug/ml) and either vehicle or 1,25D (100nM) for 6 hours. Since the mechanism of action of 1,25D is primarily transcriptional, the effects of 1,25D on cell proliferation are expected to be downstream to the transcriptional effects, hence, we chose a shorter time for the transcriptional response compared to the longer time point for cell proliferation. In addition, a strong transcriptional response to 1,25D in PBMCs had been observed at a similar time point in a previous study from our group; none of the subjects from the previous study were included here [39]. Cell type composition of the PBMCs was measured using flow cytometry as previously reported for these samples [38], where proportions of T cells including T helper cells (CD4^+^) and cytotoxic T (CD8^+^) cells, B cells, monocytes and neutrophils were measured using antibodies specifically targeting these cell types. The cell type composition and other sample characteristics are summarized in **S1 Table**.

### Cellular Proliferation Measurements

After 48 hours of treatment, cell proliferation was measured by H^3^-thymidine incorporation using standard protocols as previously described [38–40]. Briefly, proliferation measurements, obtained from the counts per minute (CPM) values of the thymidine incorporation assay, were obtained for each treatment (1,25D+PHA or vehicle+PHA). The median CPM values were taken from across the three replicates. Percent inhibition of proliferation by 1,25D (I_max_) was calculated as 1 –[(proliferation in 1,25D+PHA)/(proliferation in EtOH+PHA)], and fit to a normal distribution. The median CPM values for each treatment across all samples, and the corresponding I_max_ values are listed in **S2 Table**. Associations between covariates and I_max_ were tested using a simple linear regression.

### Transcriptional Response Profiling

After 6 hours of treatment with PHA and 1,25D or vehicle, the three replicates from each donor were pooled before RNA extraction. Total RNA was extracted from each pool with the RNeasy Plus Mini Kit (Qiagen 74134). Total RNA was reverse transcribed into cDNA, labeled, hybridized to Illumina (San Diego, CA, USA) Human HT-12 v3 Expression Beadchips and scanned at the University of Chicago Functional Genomics Core facility. We performed low-level microarray analyses using the Bioconductor software package LUMI [41] in R, as previously described [40]. Briefly, we annotated probes by mapping their sequence to RefSeq (GRCh37) transcripts using BLAT. We discarded probes that mapped to multiple genes, or contained one or more HapMap SNPs. We applied variance stabilization transformation to all arrays, discarded poor quality probes, and quantile normalized the arrays using the default method implemented in the lumiN function. After these filters, probes mapping to 11,897 genes were used in downstream analyses. The normalized gene expression values are available in **S3 Table**. We used a paired t-test to identify genes that were differentially expressed between 1,25D- and vehicle-treated samples. The results from the paired t-test analysis for all 11,897 genes are available in **S3 Table**. False-discovery rates (FDR) were estimated using the q value function in R [42]. Gene set enrichment analysis was performed using the commercially available software Ingenuity Pathway Analysis (IPA).

### Genome-Wide Association of Inhibition of Cellular Proliferation by 1,25D (I_max_)

Samples were genotyped on two Illumina Omni BeadChip platforms, with a total of 884,015 SNPs across the genome genotyped for each donor, as previously described [38]. We then imputed genotypes at all SNPs identified in the 1000 Genomes Project [43] using IMPUTE2 [44], applying the output file flag option “-pgs_miss”, which replaces the missing genotypes at typed SNPs with imputed genotypes. We filtered SNPs for minor allele frequency (>0.1), imputation quality (>0.9), and departure from Hardy Weinberg equilibrium (p> 0.001), resulting in a total of 4,047,158 SNPs available for all 88 samples.

We performed a genome-wide association scan (GWAS) of the cellular inhibition of proliferation by 1,25D (I_max_) using a likelihood ratio test correcting for genome-wide proportions of African ancestry to control for spurious associations due to population structure. Genome-wide African ancestry proportions in each donor were estimated using STRUCTURE which uses multi-locus genotype data to investigate the genetic structure of populations [45]. Prior to the GWAS analysis, I_max_ was corrected for all covariates including age, gender, and cell type proportions.

### Mapping Variation in Transcriptional Response

We performed a genome-wide test for association between log_2_ fold change at every gene and SNPs within 100kb of the transcriptional start site of each gene. Transcriptional response profile data was not collected for 3 out of the 88 donors. For the 85 donors, the total number of genome-wide SNPs available for eQTL mapping that passed the filters described earlier was 4,100,242. eQTL mapping was performed using Matrix eQTL software, which performs a linear regression test for association between each SNP and each transcript, modeling the additive linear genotype effect on transcriptional response [46]. FDRs were calculated according to the Benjamini and Hochberg method [47]. We also corrected for genome-wide African ancestry proportions in this analysis.

As a complementary approach, we applied a Bayesian statistical framework that identifies different genotype-treatment interaction patterns, using the statistical software BRIdGE [48]. We mapped interaction eQTLs within 100kb of expressed genes, modeling four conditions through which SNPs could interact with transcriptional response phenotype under the two treatment conditions (1,25D and control): (i) Control-only model, where genotype is associated with transcript levels in control-treated aliquots, but not in 1,25D-treated aliquots, (ii) 1,25D-only model, where genotype is associated with transcript levels in 1,25D-treated aliquots, but not in EtOH-treated aliquots, (iii) General interaction model, where genotype is associated with transcript levels in both conditions, but with different effects in each condition, and (iv) No interaction model, where genotype is associated with transcript levels in both conditions, with equal effect in each condition (baseline eQTLs). Using a hierarchical model, information across SNPs in each gene region and across genes was combined, and a posterior probability for each gene that it follows each of the models, and that it is affected by a SNP that follows that model, was calculated.

### Identifying eQTLs within Regulatory Regions

We reanalyzed published data sets of VDR ChIP-seq obtained in THP-1 monocytic cell lines treated with 1,25D and LPS or 1,25D alone [49], and FAIRE-seq performed in THP-1 cells treated with 1,25D [50]. First, we aligned sequence reads to the human reference (GRCh37) using BWA backtrack 0.7.5. Second, we kept only sequence reads with phred-scaled mapping quality ≥ 30 using samtools v1.1 [51]. Third, PCR duplicate were removed with picard tool v 1.130 (http://broadinstitute.github.io/picard/). For the ChIP-seq data sets, we confirmed the quality of data sets by strand cross-correlation (SCC) analysis [52] implemented in the R script “run_spp_nodups.R” packaged in phantompeakqualtools (https://code.google.com/p/phantompeakqualtools/). Statistically significant peaks were identified using MACS version 2 [53] with the following essential command line arguments: macs2 callpeak—bw X -g hs—qvalue = 0.05 -m 5 50, where X is a length of the bandwidth that was defined as a fragment length calculated by SCC for the ChIP-seq data or as 200 bp for the FAIRE-seq data reported in Seuter *et al*. (2012).

Out of the 4,100,242 SNPs available for eQTL mapping, we identified subsets of these SNPs that were within ChIP-seq and FAIRE-seq peaks. We then used these subsets of SNPs to map response eQTLs using Matrix eQTL as described in the previous section.

### Overlap between Cellular and Transcriptional Response Phenotypes

To identify genes whose transcriptional response to 1,25D may play a role in the inhibition of cell proliferation, we performed linear regression to test the association across individuals between the cellular response phenotype (I_max_), and log-fold change response (1,25D-treated over vehicle-treated expression), and we estimated FDR using the q value function in R. We also applied a Bayesian method with the program Sherlock [54] to predict putative causal genes associated with I_max_. This method predicts causal genes by identifying SNPs in these genes that are associated both with gene expression in *cis* and *trans*, and with the trait of interest, in our case, I_max_. We used the results from the response *cis*-eQTL mapping and the GWAS of I_max_ to perform this analysis, setting the prior for association of each SNP with gene expression in *cis*, as well as association of each SNP with I_max_, to 0.01. We chose this high prior due to the fact that we were examining transcriptional and cellular response phenotypes in primary cells obtained from the same individuals. The statistical significance of the Bayes factor for each gene was indicated by the corresponding p-values, which were calculated by permutation of the GWAS data, as detailed by He *et al*. (2013).

## Results

### Mapping Variation in Inhibition of Cellular Proliferation by 1,25D

To characterize inter-individual variation in cellular response to 1,25D, we measured cellular proliferation in PBMCs, which had been stimulated for 48 hours with PHA in the presence of either 1,25D or its vehicle (EtOH) as a control. I_max_ was calculated as the proportion of proliferation in 1,25D treated cells relative to proliferation in vehicle-treated cells. The average I_max_ value across the 88 samples was 25%, with a maximum I_max_ of 80%. Negative I_max_ values were observed in some samples, where there was more proliferation after 1,25D treatment (**S2 Table** and **S3 Fig**).

Using a simple linear regression, we measured the association between each donor’s age, gender, time of collection, batch, serum 25D and cortisol levels, and found no significant correlations between these covariates and I_max_. We also found no significant correlations between cell type proportions, as well as baseline levels of the vitamin D receptor gene (*VDR*), and I_max_ (**S4 Table**). However, to avoid any potential sources of confounding, we corrected I_max_ for all of these covariates before further downstream analyses.

To control for spurious associations potentially caused by population structure, we corrected for the proportion of genome-wide African ancestry in each donor, estimated using the program STRUCTURE. The median proportion of African ancestry in our donors was 81.4%, with an interquartile range of 14.7%. There were no significant correlations between I_max_, or the other covariates, and proportion of African ancestry. However, there was a negative correlation between the genome-wide proportion of African ancestry and serum 25D levels (p = 0.035, β = -0.034) (**S2 Fig**), which suggests a genetic contribution to the higher prevalence of vitamin D insufficiency observed in African Americans [24]. The average serum 25D level in our African American donors was 20.81nM with a standard deviation of 10.39nM, which is a level considered to be at risk for deficiency according to the Institute of Medicine definitions (less than 30nM) [55].

**Fig 1:**
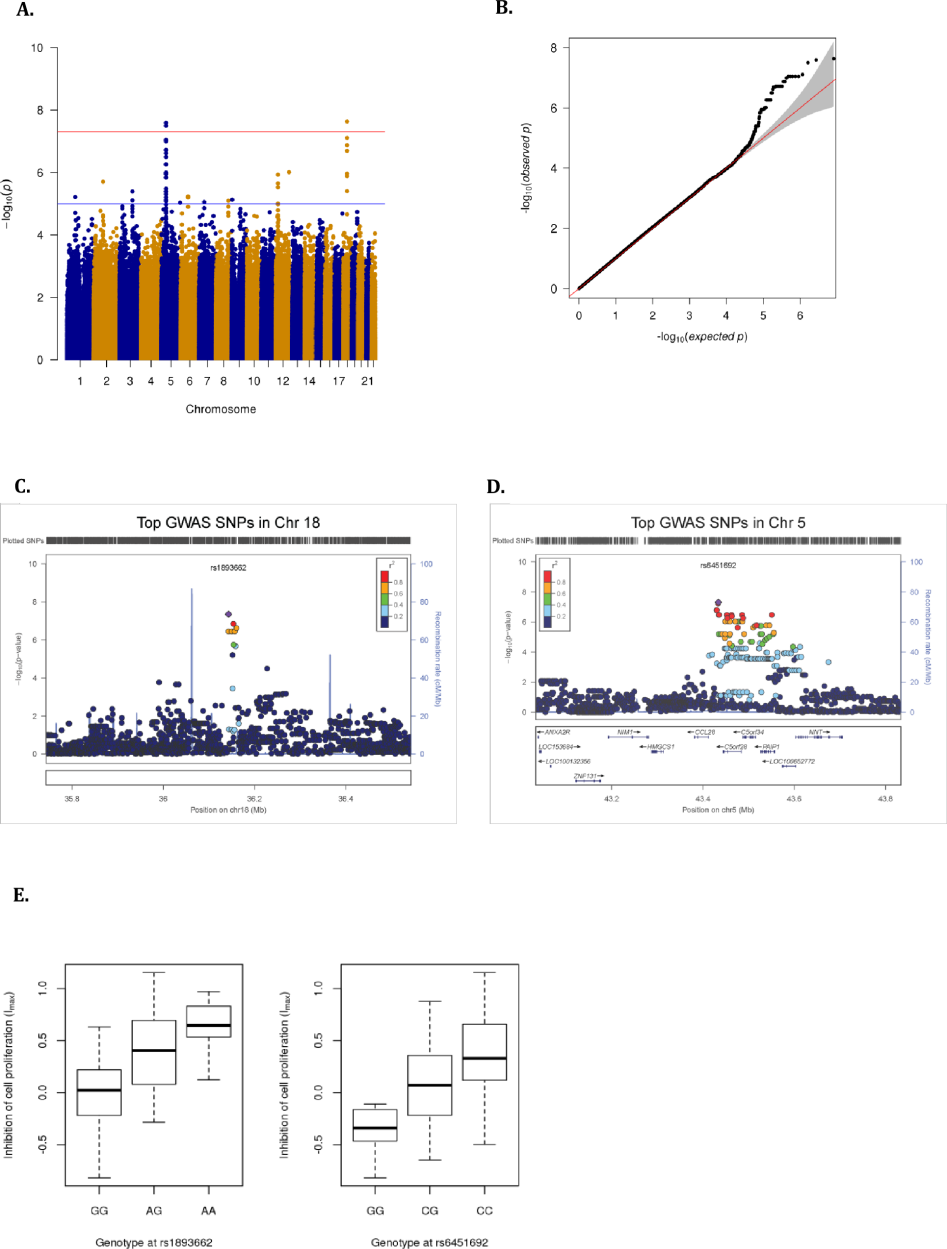
GWAS of inhibition of cellular proliferation by 1,25D (I_max_). **(A)** Manhattan plot of -log_10_ p-values of association of genome-wide variants with I_max_. **(B)** Quantile-quantile (QQ) plot of distribution of observed -log_10_ p-values on the y-axis, versus the expected -log_10_ p-values on the x-axis. LocusZoom plots of the Imax GWAS associated regions in **(C)** chromosome 18 around rs1893662, and **(D)** chromosome 5 around rs6451692 (400kb windows, using 1000 genomes African populations as a reference). **(E)** Boxplots of I_max_ relative to genotypes of rs1893662 and rs6451692. I_max_ was corrected for age, gender, time of blood collection, batch, serum 25D levels, serum cortisol levels, and cell type proportions, and then fit to a normal distribution.

To investigate the genetic bases of variation in I_max_, we carried out a genome-wide association scan for a total of 4,047,158 SNPs and identified genome-wide significant SNPs in chromosomes 5 and 18 (**Fig 1A and 1B**). The top signal of association was an intergenic SNP in chromosome 18 (rs1893662, p = 2.32 x 10^−8^) (**Fig 1A and 1C, S5 Table**). The A allele was associated with increased inhibition of proliferation (**Fig 1E**), and had a lower frequency in populations of African ancestry compared to European and Asian populations (allele frequency: 0.325, 0.811, and 0.648 respectively) (**S4A Fig**). The next strongest signal of association was an intergenic SNP in chromosome 5 (rs6451692, p = 2.55 x 10^−8^) (**Fig 1A and 1D, S5 Table**). The C allele was associated with increased inhibition of proliferation (**Fig 1F**), and had a higher frequency in populations of African ancestry compared to European and Asian populations (allele frequency: 0.839, 0.565, and 0.198 respectively) (**S4B Fig**). The closest gene to this SNP is *CCL28*, which encodes a chemokine that recruits T cells, eosinophils, and B cells to mucosal sites; other genes within 100 kb of this SNP are two uncharacterized open reading frames (*C5orf28* and *C5orf35*) and *PAIP1*, which plays a role in stimulating translation initiation. Interestingly, we observed a marginal association between rs6451692 C allele and transcriptional response of *PAIP1* to 1,25D (p = 0.02, beta = -0.39) (**S6 Table**). In addition, this SNP lies less than 1 kb away from H3K4me1 enhancer-associated chromatin marks, DNase I hypersensitive sites and binding events for transcription factors such as TCF7L2, GATA3 and CEBPB in seven cell lines from the ENCODE project, including lymphoblastoid cell lines [56] (**S5 Fig**). These chromatin marks highlight the potential regulatory activity of rs6451692 on transcriptional activity in immune cells.

To determine the proportion of variation of I_max_ explained by the top two SNPs in chromosomes 18 and 5, we examined the correlation coefficient from the linear model measuring the association between the top two associated SNPs and I_max_. These two SNPs had a large effect on I_max_, where rs1893662 explained 29.94% of the phenotypic variation in our samples, while rs6451692 explained 29.8% of the phenotypic variation in our samples. These top two SNPs explained ~45% of the variation in I_max_.

### Mapping Variation in Transcriptional Response to 1,25D

We measured the expression of 11,897 genes in PBMCs from 85 donors treated with 100nM 1,25D and vehicle for 6 hours. We identified 720 genes differentially expressed (DE) in response to 1,25D at a FDR<0.01. Biological pathways significantly enriched among these genes included immune response pathways such as TREM1 signaling (p = 4.0 x 10^−7^, FDR = 2 x 10^−4^), Granulocyte differentiation and Diapedesis (p = 2.0 x 10^−5^ FDR = 4 x 10^−3^), and T Helper Cell Differentiation (p = 6.0 x 10^−4^, FDR = 6.5 x 10^−2^) (**S7 Table**), confirming the important role of 1,25D as an immunomodulator. In addition, there was an enrichment of the VDR/RXR activation pathway (p = 7.0 x 10^−4^, FDR = 6.5 x 10^−2^), including genes such as *CD14*, which encodes a monocyte surface antigen mediating innate immune response to bacterial lipopolysaccharide (LPS), *CAMP* which encodes an antimicrobial peptide, and *CYP24A1* which encodes the enzyme that initiates the degradation of 1,25D. A previous study characterizing patterns of transcriptional response to 1,25D and LPS in primary monocytes also found an overlapping list of immune response pathways identified in this study enriched among genes that were significantly down-regulated by 1,25D [57].

In order to identify polymorphisms that influence the transcriptional response to 1,25D, we tested the association between log_2_ fold change in transcript levels at each expressed gene and SNPs within 100kb of each gene using Matrix eQTL. Because DE genes tend to be those with consistent differences in transcript levels across all individuals, they may be biased against genes with common regulatory polymorphisms. For this reason, we did not limit our mapping analyses to the DE genes. We identified response *cis*-eQTLs for 8 genes at a FDR<0.10, with the most significant response eQTLs including the transcriptional factor ets variant 3-line (*ETV3L*), and EH-domain containing 4 (*EHD4*), which plays a role in early endosomal transport (**Table 1A, S6 Fig**). Mapping log_2_-fold change does not distinguish among the types of genotype-by-treatment interactions that influence transcriptional response. To do that, we applied a Bayesian statistical framework using the BRIdGE software, which compares different interaction models to each other and to a null model of no genotypic effect in both treatment conditions. We identified 4 genes with high confidence interactions (posterior probability of interaction > 0.7) between 1,25D treatment and SNP genotype; all these interaction eQTLs followed a 1,25D-only model, namely genotype has an effect on transcript levels in the 1,25D-treated aliquot but not in the control-treated one (**Table 1B**). These interaction eQTLs included the top 2 most significant response eQTLs that had been identified by mapping log_2_ fold change: *ETV3L* and *EHD4*. In addition, we identified interaction eQTLs in leucine rich repeat containing 25 (*LRRC25*), which is involved in activation of various immune cell types, and the transcriptional regulator unkempt family zinc finger (*UNK*).

**Table 1:** *cis*-eQTLs for transcriptional response to 1,25D.

**(A) *cis*-eQTL mapping of log-fold change expression using Matrix eQTL**					
**SNP**	**Gene**	**T-Statistic**	**P-value**	**FDR**	**Beta**
rs74116976	*ETV3L*	6.77	1.73 x 10^−09^	1.17 x 10^−4^	0.84
rs11070354	*EHD4*	6.11	3.16 x 10^−8^	1.28 x 10^−3^	0.86
rs7311057	*PARPBP*	5.73	1.56 x 10^−7^	2.43 x 10^−2^	0.79
rs59937851	*ZNHIT1*	5.41	5.98 x 10^−7^	1.52 x 10^−2^	1.02
rs7178702	*SPESP1*	5.12	1.97 x 10^−6^	1.30 x 10^−2^	0.69
rs10282056	*COBL*	-4.78	7.50 x 10^−6^	3.10 x 10^−2^	-0.92
rs62014366	*VWA9*	4.74	8.66 x 10^−6^	4.67 x 10^−2^	0.94
rs7779605	*CPED1*	4.31	4.38 x 10^−5^	7.63 x 10^−2^	0.75
**(B) Interaction *cis*-eQTL mapping using BRIdGE**					
**Gene**	**SNP**	**Posterior probability for each interaction model**			
		**Control-only**	**1,25D-only**	**General interaction**	**No interaction**
*EHD4*	rs1648856	0	0.994	0	0.001
*LRRC25*	rs3848646	0	0.965	0	0.027
*UNK*	rs8081606	0	0.803	0	0.049
*ETV3L*	rs6689823	0	0.723	0	0.277

**Fig 2:**
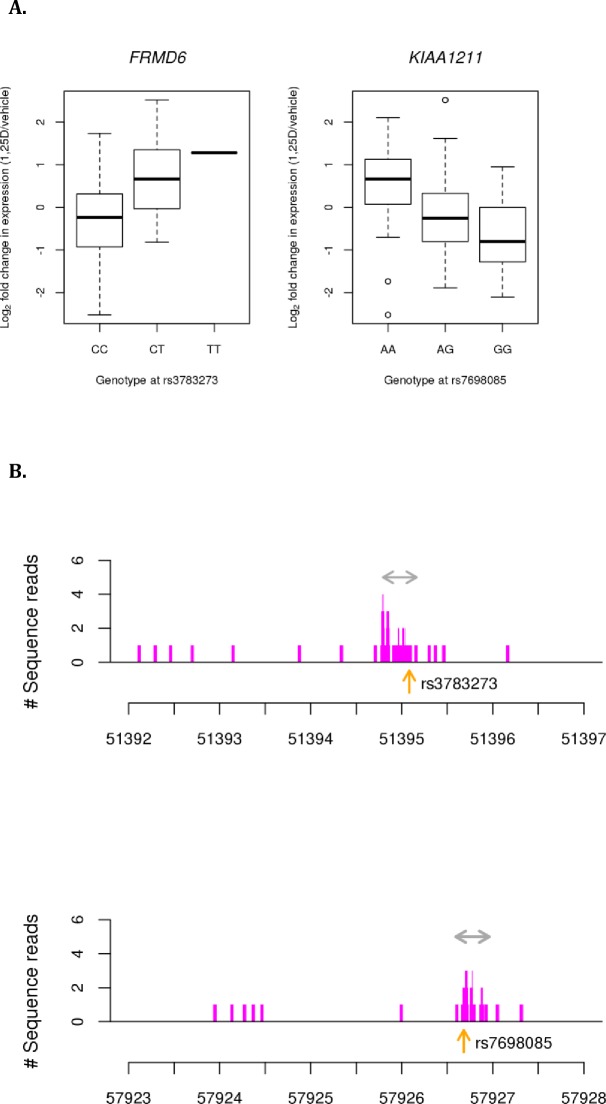
Associations between SNPs in vitamin D receptor (VDR) binding sites and transcriptional response. (**A**) Boxplots showing the effect of genotype on log_2_ fold change of *FRMD6* and *KIAA1211* transcript levels, with genotypes of the associated SNPs on the x-axis. (**B**) Location of SNPs associated with transcription response of *FRMD6* (rs3783273, top panel) and *KIAA1211* (rs7698085, bottom panel) within VDR binding sites, indicated by the gray horizontal arrows. The SNP locations are indicated by the vertical orange arrows. VDR binding site information was obtained from a published ChIP-seq dataset from THP-1 monocytic cells treated with 1,25D and bacterial lipopolysaccharide (LPS).

To evaluate additional response *cis*-eQTLs found in VDR response elements, we identified 988 SNPs within VDR ChIP-seq peaks from a dataset of published THP-1 monocytic cell lines treated with 1,25D [49], and mapped response eQTLs using this subset of SNPs. At a distance of 1Mb, we identified statistically significant response eQTLs (FDR < 0.10) in two genes: FERM Domain Containing 6 (*FRMD6*), a key activator of the Hippo kinase pathway with important roles in regulating cell proliferation and apoptosis [58], and the undefined *KIAA1211* (**Fig 2**). In addition, we identified 17,417 SNPs within open chromatin regions, identified by FAIRE-seq from a published dataset of THP-1 monocytic cell lines treated with 1,25D [50]. Within this subset, we identified statistically significant response eQTLs (FDR < 0.10) in *ETV3L*, *EHD4* and *ZNHIT1* (**S8 Table**). These eQTLs were in strong linkage disequilibrium (LD) with the response eQTLs we had identified for the same genes (r^2^ = 0.93, 0.69 and 0.95 for *ETV3L*, *EHD4* and *ZNHIT1*, respectively), raising the possibility that these response eQTLs are due to variants affecting open chromatin conformation.

### Combined Analysis of Cellular and Transcriptional Response Phenotypes

We examined the relationship between the two 1,25D response phenotypes: transcriptional response and the inhibition of cellular proliferation. To evaluate whether the SNPs associated with inhibition of cellular proliferation exerted their effects through regulation of transcriptional response, we first examined associations between the two most significant I_max_ GWAS SNPs and log_2_ fold change expression at all 11,897 genes expressed in the PBMCs. At a FDR < 0.10, we found no statistically significant associations. We then focused on the subset of genes where log_2_ fold change in expression was associated with I_max_, reasoning that these genes are more likely to share genetic variation influencing both transcriptional response and inhibition of cell proliferation. Using a linear regression approach, we identified 16 associated genes at an FDR < 0.2 (**S9 Table**). When we considered only these genes, we found significant associations between two I_max_-associated genes (*PCSK6* and *RASL11A*) and the top GWAS SNP in chromosome 18, rs1893662, and one I_max_-associated gene (*KNCN*) with the second GWAS SNP in chromosome 5, rs6451692 (**Table 2**), at a Bonferroni-corrected p < 3.125 x 10^−3^. Both *PCSK6* and *KNCN* are involved in vesicular trafficking and secretory pathways, highlighting potential molecular mechanisms involved in inhibition of proliferation by vitamin D.

**Table 2:** Association between top I_max_ GWAS SNPs and transcriptional response.

	rs1893662		rs6451692	
Gene Name	Beta	P-value	Beta	P-value
*PCSK6*	-2.09	**2.1 x 10**^**−3**^	-0.78	0.26
*SMARCD3*	2.01	**2.1 x 10**^**−3**^	0.93	0.16
*RASL11A*	2.08	**2.3 x 10**^**−3**^	1.17	0.09
*KNCN*	-1.24	3.21 x 10^−2^	-1.85	**9.95 x10**^**-4**^

We further predicted putative causal genes associated with I_max_ based on a Bayesian approach implemented in the program, Sherlock, using our response eQTL and GWAS of I_max_ data. At p < 10^−4^ (FDR = 0.3), we identified three putative I_max_-associated genes, including the translation initiation gene *PAIP1*, a transcriptional repressor gene *ZNF649*, and a golgin family gene *GORAB* (**S10 Table**). Interestingly, the top I_max_-associated SNP in chromosome 5, rs6451692, was identified as being associated with transcriptional response of *PAIP1* using this method, which suggests that this SNP influences the inhibition of cell proliferation through a transcriptional mechanism in PBMCs.

## Discussion

While the inter-individual variation in the circulating inactive form of vitamin D, 25D, has been well documented, little is known about the inter-individual variation in immune response to the active 1,25D. In this study, we identified several variants underlying variation in response to 1,25D both at the cellular and transcriptional level using primary peripheral blood mononuclear cells from a cohort of healthy individuals of African-American ancestry. These variants highlight genes with an important role in mediating the immunomodulatory effects of 1,25D, thereby providing a genetic basis for inter-individual variation in those aspects of the immune response influenced by vitamin D.

Intergenic SNPs in chromosome 5 that were significantly associated with inhibition of cellular proliferation by 1,25D are located close to several genes such as *CCL28*, which encodes a chemokine that recruits T cells, eosinophils, and B cells to mucosal sites [59–61], and *PAIP1* which encodes a protein that interacts with poly(A)-binding protein and with the eIF4A cap-binding complex, stimulating translation initiation [62]. Interestingly, we found a marginal association between rs6451692 and down-regulation of *PAIP1*, raising the possibility that this polymorphism influences the inhibitory effects of 1,25D on immune cell proliferation by regulating the transcriptional response of a translation initiation gene.

We observed several regulatory marks near rs6451692 in seven cell lines from the ENCODE project, including an enrichment of H3K4me1 histone mark, which is associated with enhancers. There was also an abundance of transcription factor binding events in this region, where rs6451692 overlaps a TCF7L2 binding site. TCF7L2 is a member of the high mobility group DNA binding protein family of transcription factors which has been implicated in type 2 diabetes risk [63–65]. Other transcription factors with binding sites in the region include RXRA, which binds to the VDR, forming a heterodimer which then regulates transcription of vitamin D-responsive genes, GATA3 which has important roles in T cell development [66, 67], and CEBPB which plays an important role in regulating immune and inflammatory response genes [68–71]. The abundance of transcription factor binding events in this region suggests that the regulatory activity of rs6451692 on the surrounding genes could involve enhancer activity. Further functional validation assays specifically in PBMCs treated with vitamin D are needed to elucidate the regulatory mechanisms of this I_max_ GWAS interval.

In addition, from the Genotype-Tissue Expression (GTEx) project catalogue [72], we observed that rs6451692 is associated with variation in transcript levels of surrounding genes in multiple tissues. The C allele is associated with decreased expression of *CCL28* in the pancreas, decreased expression of *NNT* in skeletal muscle, and decreased expression of the novel antisense long non-coding RNA *RP11-159F24*.*5* in multiple tissues such as subcutaneous adipose, tibial nerve, testis, thyroid and skin, suggesting that this variant influences the regulation of several genes in that genomic region. *RP11-159F24*.*5* was not covered by probes in our expression microarrays, therefore we cannot determine if rs6451692 has effects on the expression of this gene in PBMCs.

Enrichment of immune response pathways such as TREM1 signaling, which enhances innate immune responses to microbial infections and activates pro-inflammatory responses [73], and T helper cell differentiation among the genes that respond transcriptionally to 1,25D, underscores the important immunomodulatory role played by 1,25D [8–10]. This is consistent with results from previous studies from our group investigating the transcriptional response to 1,25D in PBMCs [39] and transcriptional response to 1,25D and bacterial lipopolysaccharide (LPS) in primary monocytes [57]. Both of these studies found an enrichment of immune response pathways, particularly among genes that were down-regulated by 1,25D. This highlights the important immunomodulatory role played by 1,25D across cells in both the innate and adaptive immune system. In addition, translation initiation pathways were significantly enriched among the genes that were up-regulated by 1,25D in monocytes in the previous study. This pathway was not significantly enriched amongst the DE genes in PBMCs in this study (**S7 Table**), which could indicate that this effect is particularly strong in the innate immune cells. It was however interesting to note the marginal association between one of the top I_max_ SNPs, rs6451692, and transcriptional response of *PAIP1*, a translation initiation gene.

Several studies have mapped genome-wide VDR binding sites in different immune cell lines [49, 74, 75]. Interestingly, one study examined VDR binding sites in primary CD4^+^ T cells from nine individuals with varying 25D levels and reported a correlation between 25D levels and number of VDR binding sites [76], directly supporting the notion that vitamin D status affects the response to vitamin D. In addition, genome-wide maps of VDR binding sites allow identification of genetic variants within VDR binding sites that in turn may influence variation in the transcriptional response to vitamin D. Interestingly, one such study reported that many risk variants for autoimmune diseases detected in genome-wide association studies fall within VDR binding sites [70], suggesting that disease risk is influenced not only by inter-individual variation in 25D levels, but also by variation in the response to vitamin D. To build on these studies, we focused on mapping variants that regulate genome-wide transcriptional response to 1,25D in primary PBMCs. The *cis*-response eQTLs identified in this study highlighted several genes that could play an important role in mediating the effects of 1,25D in the immune response. Genes identified using both the linear regression and Bayesian eQTL mapping approaches included *ETV3L*, which is a transcriptional regulator that has been reported to play a role in inhibiting proliferation of neural progenitor cells [77], and *EHD4*, which plays a role in controlling early endosomal trafficking [78, 79]. Furthermore, we identified statistically significant response eQTLs in regions of open chromatin, marked by FAIRE-seq peaks in *ETV3L*, *EHD4*, and *ZNHIT1*—a gene that is implicated in regulating the transcriptional activity of the orphan nuclear receptor Rev-erbbeta [80]. Interestingly, both *ETV3L* and *ZNHIT1* are transcriptional regulators, raising the possibility that these loci could play a role in modulating transcriptional response of other genes to 1,25D in immune cells.

We then identified variants within VDR binding sites that regulate transcriptional response possibly by altering the structure or accessibility of the VDR binding site. We did this by combining our *cis*-response eQTL data with a published VDR ChIP-seq dataset from a monocytic cell line [49]. We identified a response eQTL within a VDR binding site in *FRMD6*, which is part of the conserved Hippo pathway playing a critical role in controlling organ size by regulating both cell proliferation and apoptosis [81, 82]. *FRMD6* has been linked to various complex diseases such as asthma, Alzheimer’s disease, and lung cancer [82–84], where it is thought to have tumor suppressor properties. The T allele of rs3783273, which is associated with increased *FRMD6* expression (**Fig 2**), could alter the binding properties of the VDR to its receptor elements in *FRMD6* and could affect the transcriptional response of this gene to 1,25D. Given its putative tumor suppressor properties, *FRMD6* may play a crucial role in mediating the role of 1,25D in inhibiting proliferation of immune cells.

The published VDR ChIP-seq and FAIRE-seq data were collected in a monocytic cell line stimulated with LPS and treated with 1,25D. Our data, in contrast, were collected in a heterogeneous population of immune cells treated with PHA, which was shown to stimulate transcriptional responses related to both innate and adaptive immunity [38]. Therefore, the data from the monocytic cell line may miss some peaks specific to other cell types present in PBMCs, that is, primarily T cells, but it may also contribute valuable information about the regulatory architecture of 1,25D response in immune cells. Future work should incorporate VDR ChIP-seq and FAIRE-seq studies in primary immune cells under the same experimental conditions.

Using both simple linear regression analysis and a Bayesian approach, we combined the information from response *cis*-eQTL mapping and the GWAS of I_max_ to identify candidate genes mediating the inhibitory effects of cellular proliferation by 1,25D. Genes such as *PAIP1*, *ZNF649* and *GORAB* contained I_max_-associated SNPs that also regulated transcriptional response of these genes in *cis*. While *PAIP1* encodes a protein that is involved in initiating translation, *ZNF649* encodes a transcriptional repressor that inhibits transcription factor complexes such as AP-1 which is involved in cellular proliferation and survival [85–87], and *GORAB* encodes a golgin family member with roles in the intracellular membrane trafficking and the secretory pathways of the Golgi apparatus [88, 89]. In addition, we identified *trans* effects of the top GWAS SNPs on transcriptional response of genes such as *PCSK6* and *KNCN*, which both have roles in vesicular trafficking and secretory pathways, highlighting potential molecular mechanisms involved in the anti-proliferative activity of 1,25D. Increased *PCSK6* expression has been previously implicated in risk for rheumatoid arthritis [90]. Interestingly, knockdown of *PCSK6* by RNA interference significantly decreased proliferation, invasion, and migration of cultured rheumatoid arthritis synovial fibroblasts. It is plausible that the top I_max_-associated SNP, rs1893662, regulates the anti-proliferative activity of 1,25D by regulating *PCSK6* transcription in immune cells. The potential mechanisms through which these putative I_max_-associated candidate genes could mediate the inhibition of proliferation of immune cells by 1,25D should be further studied.

In summary, mapping response to 1,25D at both the cellular and transcriptional level in immune cells enabled identification of variants which may influence inter-individual variation in response to 1,25D, and identification of genes with potentially crucial roles in mediating the immunomodulatory role of 1,25D. Characterizing these genetic mediators of 1,25D activity in the immune system could inform additional therapeutic targets and markers for immune-related diseases in future randomized VD supplementation trials.

## Supporting Information

S1 Fig Experimental Design. Peripheral blood mononuclear cells (PBMCs) were obtained from 88 healthy African American donors. PBMCs were cultured for 6 hours with phytohemagglutinin (PHA) and either vehicle (EtOH) or 1,25-dihydroxyvitamin D3 (1,25D), and RNA was extracted for gene expression measurements. PBMCs from the same samples were also cultured for 48 hours with PHA and either vehicle or 1,25D for cell proliferation measurements. DNA was also extracted from PBMCs for genotyping.
(DOCX)

S2 Fig Correlation between serum 25D levels and African ancestry proportions. Serum levels of 25D are negatively correlated with global proportions of African ancestry. Serum 25D levels were corrected for age and batch effects.
(DOCX)

S3 Fig Distribution of raw counts per minute (CPM) across genotypes at the top Imax GWAS SNPs. rs1893662 is at the top panel, and rs6451692 is at the bottom panel. Boxplots of CPM in 1,25D treatment, vehicle control, and in the ratio of 1,25D to vehicle, are colored in blue, pink and green respectively.
(DOCX)

S4 Fig Global distribution of allele frequencies. The allele frequency distribution across global populations of the top I_max_ GWAS SNPs, (A) rs1893662 and (B) rs6451692. Image obtained from the Geography of Genetic Variants (GGV) browser [91].
(DOCX)

S5 Fig Magnified view of the I_max_ GWAS interval in chromosome 5. The location of rs6451692 is highlighted by the blue rectangle. Nearby enhancer marks (H3K4me1), DNase I hypersensitive sites, and transcription factor binding sites were obtained from seven cell lines from the ENCODE project [56].
(DOCX)

S6 Fig Results from 1,25D response *cis*-eQTL mapping using Matrix eQTL [46]. Boxplots show the effect of genotype on log_2_ fold change in transcript levels of 8 genes, with genotype coded as the number of copies of the minor allele. All SNPs are within 100kb of the transcriptional start site (TSS) of their respective genes.
(DOCX)

S1 Table Subject characteristics, sample collection times and cell type composition. (XLSX)

S2 Table Cell proliferation measurements. Counts per minute (CPM) measurements in 1,25D treatment and in vehicle control, and Imax values for all samples.
(XLSX)

S3 Table Gene expression values and differential expression analysis results. Normalized log_2_-expression values per individual and per treatment for 11,897 genes, and differential expression results from paired t-test comparing expression in 1,25D-treated samples to expression in vehicle-treated samples.
(XLSX)

S4 Table Correlation between I_max_ and covariates. The association between I_max_ and sample covariates was tested using a simple linear regression, indicating that there were no significant associations between Imax and the covariates (p > 0.05).
(DOCX)

S5 Table The top SNPs identified in the GWAS of I_max_. The SNPs shown have p-values < 1 x 10^−8^, which corresponds to a FDR of 0.036.
(DOCX)

S6 Table Association between top I_max_-associated SNPs in chromosome 5, and transcription response of nearby genes (within 100kb). (DOCX)

S7 Table Gene set enrichment analysis of significantly differentially expressed (DE) genes (FDR < 0.01), using Ingenuity Pathway Analysis (IPA) software. Enriched pathways shown are at a p-value threshold of 0.05. The top 8 pathways were statistically significant at a FDR < 0.10. B-H p-value* = Benjamini-Hochberg multiple testing corrected p-value.
(DOCX)

S8 Table Response *cis*-eQTLs found in open chromatin regions detected by FAIRE-seq. These response eQTLs are significant at a FDR < 0.10. The strength of the FAIRE-seq peaks is indicated by the Peak p-values and FDR, as well as the Peak fold enrichment values.
(DOCX)

S9 Table Genes whose transcription responses are associated with I_max_ at a FDR<0.2. (DOCX)

S10 Table Putative Imax-associated genes. Genes associated with I_max_ were detected using a Bayesian approach implemented in the statistical program Sherlock [54]. The strength of the association between the genes and I_max_ is given by the overall log_10_ of Bayes factor (LBF).
(DOCX)

S11 Table List of loci highlighted in this study, and their respective phenotypic associations reported in the GWAS catalogue [92]. (DOCX)
