# Supplementary material for: Mapping Variation in Cellular and Transcriptional Response to 1,25-Dihydroxyvitamin D3 in Peripheral Blood Mononuclear Cells
Source: PLoS One. 2016 Jul 25;11(7):e0159779. doi: 10.1371/journal.pone.0159779 (PMC4959717; doi:10.1371/journal.pone.0159779)

**S1 Fig. Experimental Design.** Peripheral blood mononuclear cells (PBMCs) were obtained from 88 healthy African American donors. PBMCs were cultured for 6 hours with phytohemagglutinin (PHA) and either vehicle (EtOH) or 1,25-dihydroxyvitamin D3 (1,25D), and RNA was extracted for gene expression measurements. PBMCs from the same samples were also cultured for 48 hours with PHA and either vehicle or 1,25D for cell proliferation measurements. DNA was also extracted from PBMCs for genotyping.


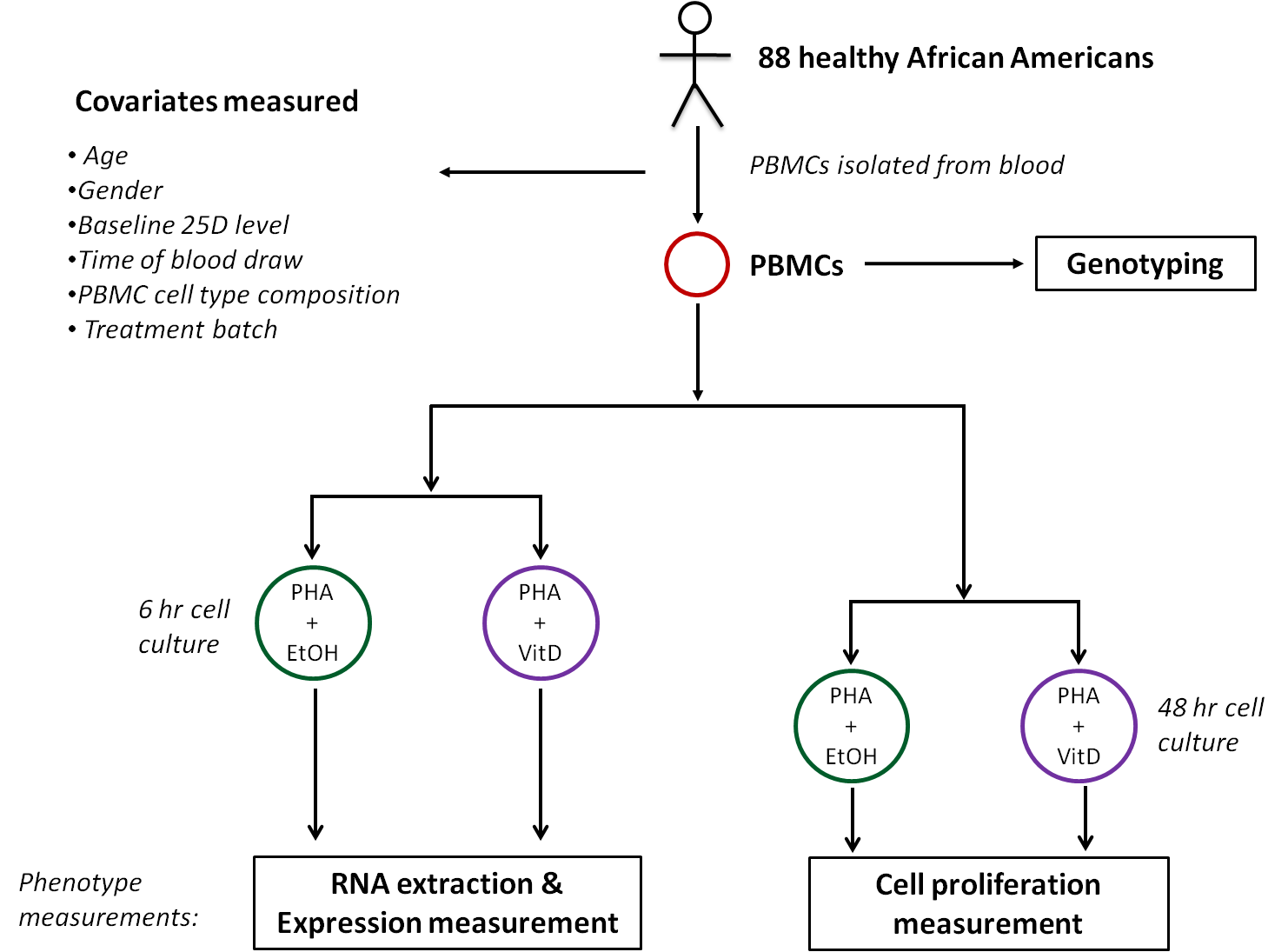

Supplement: S1 Fig — Peripheral blood mononuclear cells (PBMCs) were obtained from 88 healthy African American donors. PBMCs were cultured for 6 hours with phytohemagglutinin (PHA) and either vehicle (EtOH) or 1,25-dihydroxyvitamin D3 (1,25D), and RNA was extracted for gene expression measurements. PBMCs from the same samples were also cultured for 48 hours with PHA and either vehicle or 1,25D for cell proliferation measurements. DNA was also extracted from PBMCs for genotyping. (DOCX) [file pone.0159779.s001.docx]
