# Supplementary material for: Mapping Variation in Cellular and Transcriptional Response to 1,25-Dihydroxyvitamin D3 in Peripheral Blood Mononuclear Cells
Source: PLoS One. 2016 Jul 25;11(7):e0159779. doi: 10.1371/journal.pone.0159779 (PMC4959717; doi:10.1371/journal.pone.0159779)
Supplement: S2 Fig — Serum levels of 25D are negatively correlated with global proportions of African ancestry. Serum 25D levels were corrected for age and batch effects. (DOCX) [file pone.0159779.s002.docx]

**S2 Fig.** Serum levels of 25D are negatively correlated with global proportions of African ancestry. Serum 25D levels were corrected for age and batch effects.

**
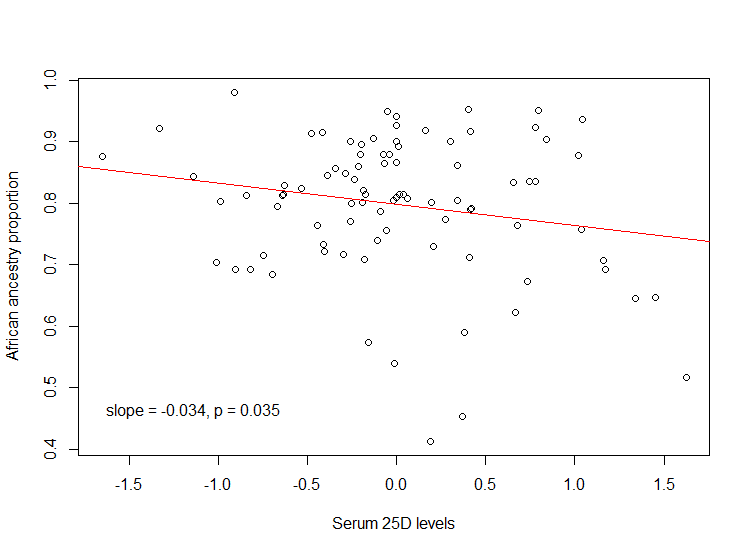
**
