# Supplementary material for: Mapping Variation in Cellular and Transcriptional Response to 1,25-Dihydroxyvitamin D3 in Peripheral Blood Mononuclear Cells
Source: PLoS One. 2016 Jul 25;11(7):e0159779. doi: 10.1371/journal.pone.0159779 (PMC4959717; doi:10.1371/journal.pone.0159779)
Supplement: S3 Fig — rs1893662 is at the top panel, and rs6451692 is at the bottom panel. Boxplots of CPM in 1,25D treatment, vehicle control, and in the ratio of 1,25D to vehicle, are colored in blue, pink and green respectively. (DOCX) [file pone.0159779.s003.docx]

**S3 Fig. Distribution of raw counts per minute (CPM) across genotypes at the top I_max_ GWAS SNPs.** rs1893662 is at the top panel, and rs6451692 is at the bottom panel. Boxplots of CPM in vehicle control, 1,25D treatment, and in the ratio of 1,25D to vehicle, are colored in pink, blue and green respectively.

**
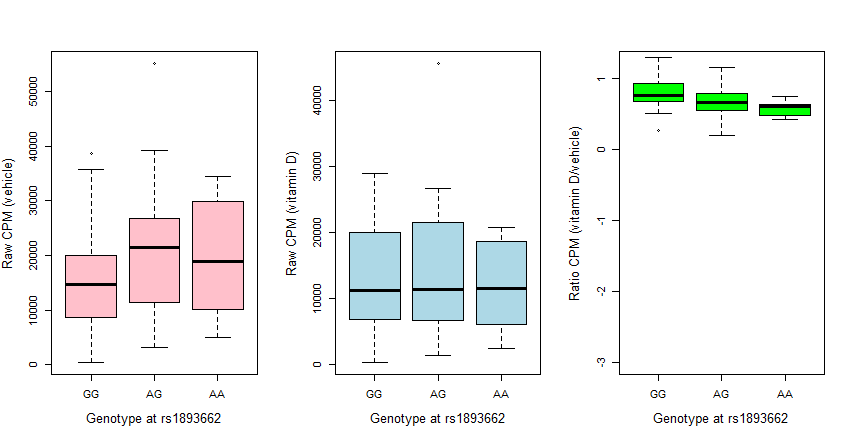
**

**
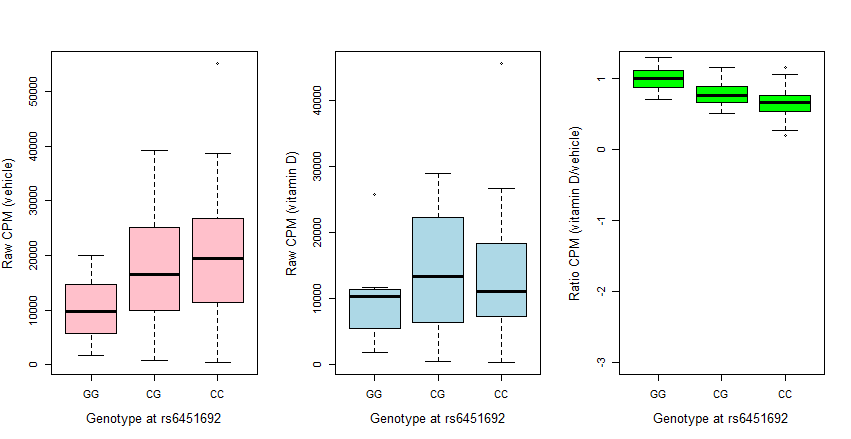
**
