# Supplementary material for: Mapping Variation in Cellular and Transcriptional Response to 1,25-Dihydroxyvitamin D3 in Peripheral Blood Mononuclear Cells
Source: PLoS One. 2016 Jul 25;11(7):e0159779. doi: 10.1371/journal.pone.0159779 (PMC4959717; doi:10.1371/journal.pone.0159779)

**S4 Fig.** Global distribution of allele frequencies across populations, at rs1893662 (**A**) and rs6451692 (**B**). Image obtained from the Geography of Genetic Variants (GGV) browser: http://www.popgen.uchicago/ggv/.


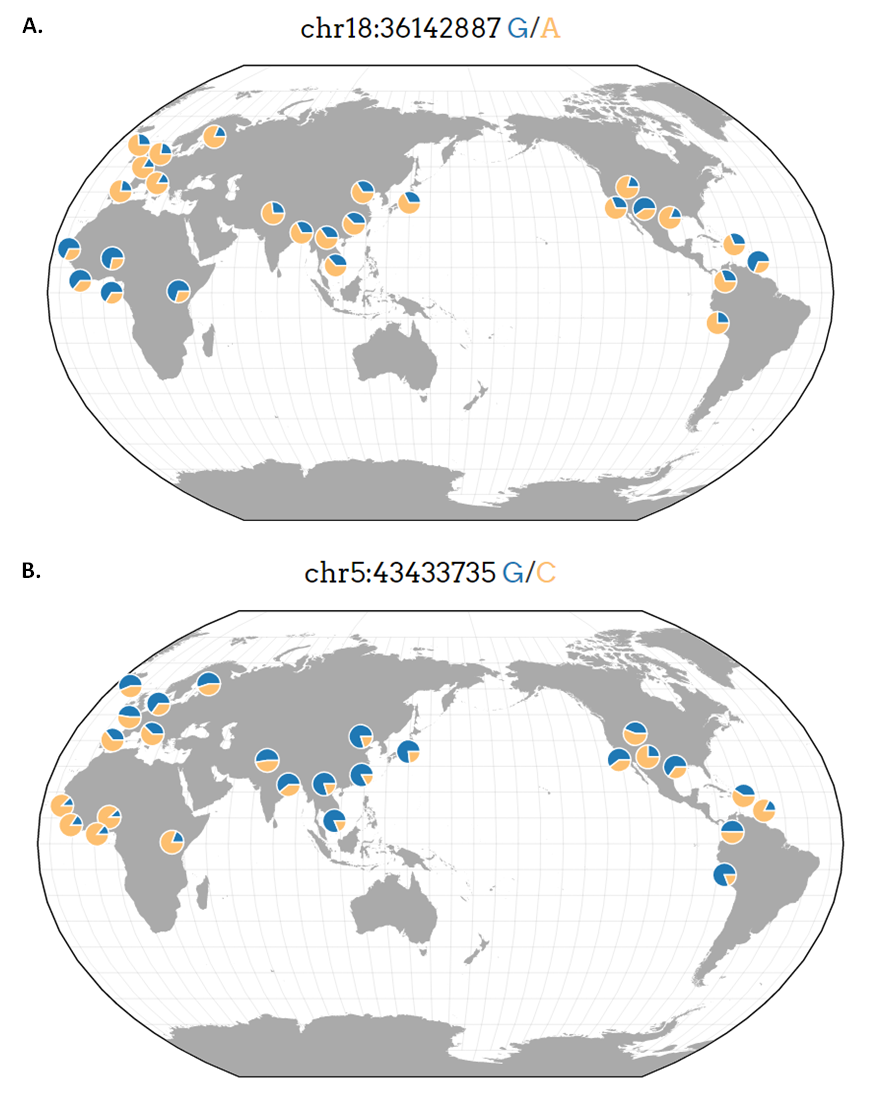

Supplement: S4 Fig — The allele frequency distribution across global populations of the top Imax GWAS SNPs, (A) rs1893662 and (B) rs6451692. Image obtained from the Geography of Genetic Variants (GGV) browser [91]. (DOCX) [file pone.0159779.s004.docx]
