# Supplementary material for: Mapping Variation in Cellular and Transcriptional Response to 1,25-Dihydroxyvitamin D3 in Peripheral Blood Mononuclear Cells
Source: PLoS One. 2016 Jul 25;11(7):e0159779. doi: 10.1371/journal.pone.0159779 (PMC4959717; doi:10.1371/journal.pone.0159779)

**S5 Fig. Magnified view of the I_max_ GWAS interval in chromosome 5.** The location of rs6451692 is highlighted by the blue rectangle. Nearby enhancer marks (H3K4me1), DNase I hypersensitive sites, and transcription factor binding sites were obtained from seven cell lines from the ENCODE project .


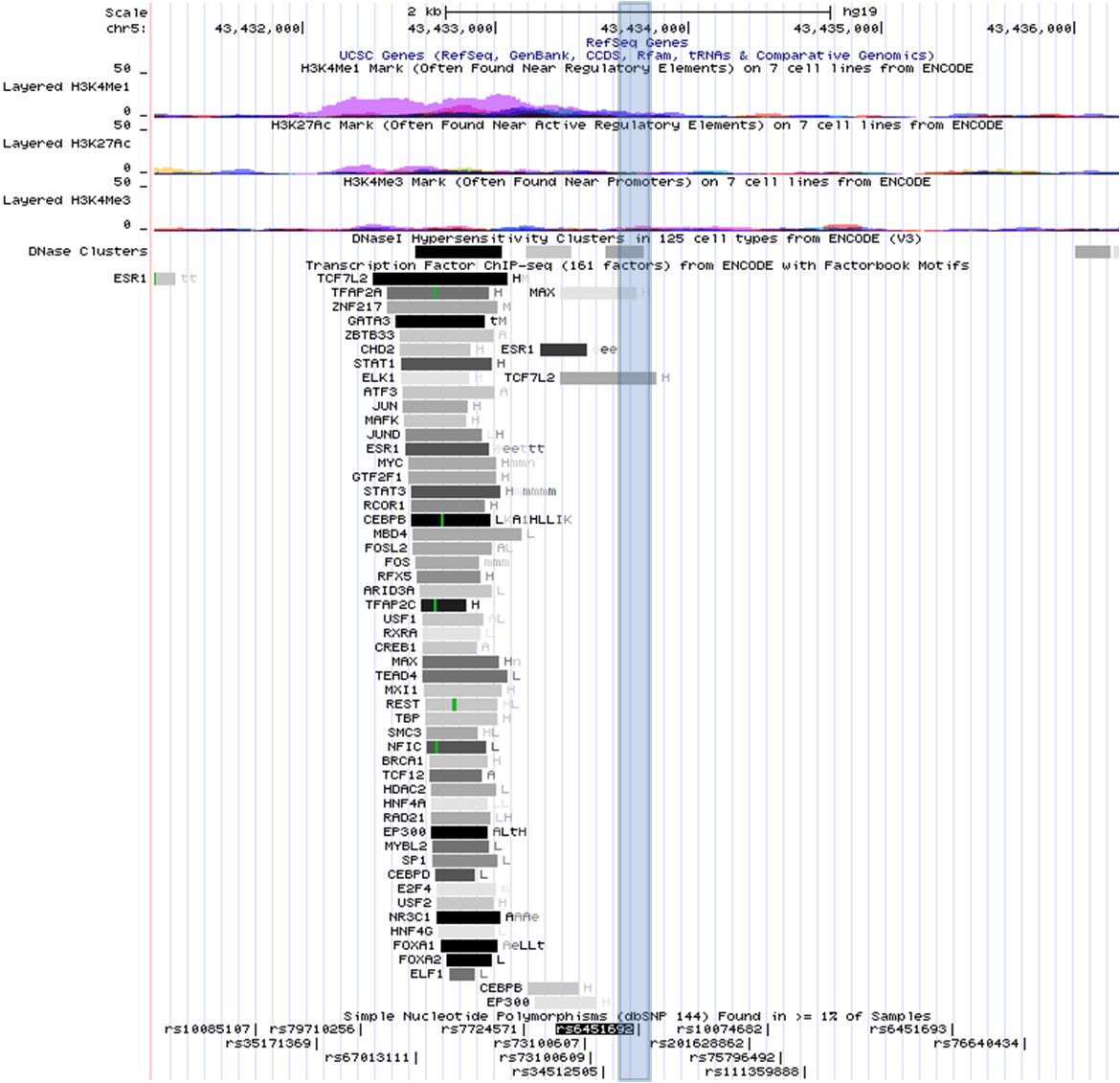

Supplement: S5 Fig — The location of rs6451692 is highlighted by the blue rectangle. Nearby enhancer marks (H3K4me1), DNase I hypersensitive sites, and transcription factor binding sites were obtained from seven cell lines from the ENCODE project [56]. (DOCX) [file pone.0159779.s005.docx]
