# Supplementary material for: Mapping Variation in Cellular and Transcriptional Response to 1,25-Dihydroxyvitamin D3 in Peripheral Blood Mononuclear Cells
Source: PLoS One. 2016 Jul 25;11(7):e0159779. doi: 10.1371/journal.pone.0159779 (PMC4959717; doi:10.1371/journal.pone.0159779)

**S6 Fig. Results from 1,25D response *cis*-eQTL mapping using Matrix eQTL**. Boxplots show the effect of genotype on log_2_-fold change in transcript levels of 8 genes, with genotype coded as the number of copies of the minor allele. All SNPs are within 100kb of the transcriptional start site (TSS) of their respective genes.


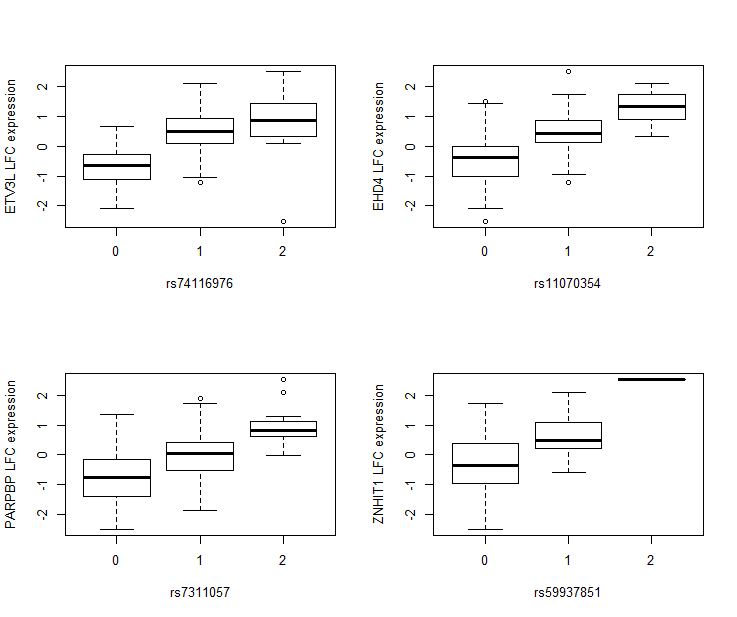


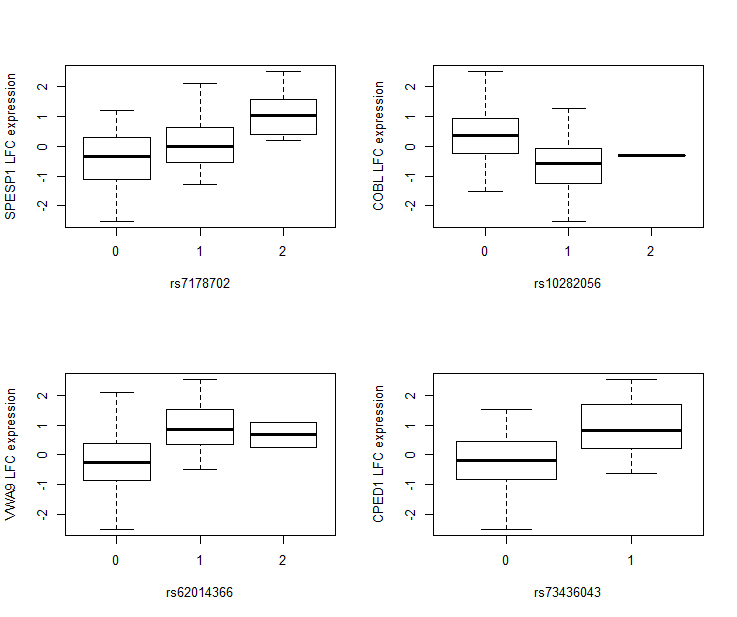

Supplement: S6 Fig — Boxplots show the effect of genotype on log2 fold change in transcript levels of 8 genes, with genotype coded as the number of copies of the minor allele. All SNPs are within 100kb of the transcriptional start site (TSS) of their respective genes. (DOCX) [file pone.0159779.s006.docx]
