# Supplementary material for: Mapping Variation in Cellular and Transcriptional Response to 1,25-Dihydroxyvitamin D3 in Peripheral Blood Mononuclear Cells
Source: PLoS One. 2016 Jul 25;11(7):e0159779. doi: 10.1371/journal.pone.0159779 (PMC4959717; doi:10.1371/journal.pone.0159779)
Supplement: S4 Table — The association between Imax and sample covariates was tested using a simple linear regression, indicating that there were no significant associations between Imax and the covariates (p > 0.05). (DOCX) [file pone.0159779.s010.docx]

**S4 Table. Correlation between I_max_ and covariates.** The association between I_max_ and sample covariates was tested using a simple linear regression, indicating that there were no significant associations between Imax and the covariates (p > 0.05)

| **Cell type** | **beta** | **p-value** |
| --- | --- | --- |
| Monocytes | 0.012 | 0.936 |
| B cells | -0.009 | 0.932 |
| T cells | -0.139 | 0.355 |
| CD8^+^ T cells | -0.104 | 0.489 |
| CD4^+^ T cells | 0.096 | 0.408 |
| Ratio CD4^+^ T cells / CD8^+^ T cells | 0.077 | 0.515 |
| Neutrophils | 0.076 | 0.521 |
| Serum 25D | 0.065 | 0.692 |
| Serum Cortisol | 0.156 | 0.161 |
| Age | -0.019 | 0.855 |
| Gender | 0.054 | 0.807 |
| Time | -0.121 | 0.264 |
| Batch | -0.014 | 0.343 |
| African ancestry proportions | -0.062 | 0.548 |
| Baseline VDR expression | -0.080 | 0.468 |
