# Supplementary material for: Mapping Variation in Cellular and Transcriptional Response to 1,25-Dihydroxyvitamin D3 in Peripheral Blood Mononuclear Cells
Source: PLoS One. 2016 Jul 25;11(7):e0159779. doi: 10.1371/journal.pone.0159779 (PMC4959717; doi:10.1371/journal.pone.0159779)
Supplement: S5 Table — The SNPs shown have p-values < 1 x 10^−8^, which corresponds to a FDR of 0.036. (DOCX) [file pone.0159779.s011.docx]

**S5 Table.** **The top SNPs identified in the GWAS of I_max_.** The SNPs shown have p-values < 10^-8^, which corresponds to a FDR of 0.036.

| **SNP** | **Chr** | **MAF** | **Nt position** | **Beta** | **P** |
| --- | --- | --- | --- | --- | --- |
| rs1893662 | 18 | 0.318 | 36142887 | 0.355 | 2.32x10^-8^ |
| rs6451692 | 5 | 0.778 | 43433735 | 0.361 | 2.55x10^-8^ |
| rs7724571 | 5 | 0.761 | 43433143 | 0.355 | 3.15x10^-8^ |
| rs4800030 | 18 | 0.318 | 36153493 | 0.352 | 7.78x10^-8^ |
| rs7707976 | 5 | 0.773 | 43429523 | 0.347 | 9.10x10^-8^ |
| rs7708443 | 5 | 0.773 | 43429761 | 0.347 | 9.10x10^-8^ |
| rs7708369 | 5 | 0.773 | 43429964 | 0.347 | 9.10x10^-8^ |
| rs750582 | 5 | 0.773 | 43430273 | 0.347 | 9.10x10^-8^ |
| rs750584 | 5 | 0.773 | 43430406 | 0.347 | 9.10x10^-8^ |
