# Supplementary material for: Mapping Variation in Cellular and Transcriptional Response to 1,25-Dihydroxyvitamin D3 in Peripheral Blood Mononuclear Cells
Source: PLoS One. 2016 Jul 25;11(7):e0159779. doi: 10.1371/journal.pone.0159779 (PMC4959717; doi:10.1371/journal.pone.0159779)
Supplement: S6 Table — (DOCX) [file pone.0159779.s012.docx]

**S6 Table. Association between top I_max_-associated SNPs in chromosome 5, and transcription response of nearby genes (within 100kb).**

| **SNP** | **Gene Name** | **Response eQTL**  **P-value** | **Beta** | I_max_ **GWAS P-value** | **SNP-Gene distance (bp)** |
| --- | --- | --- | --- | --- | --- |
| rs6451692 | *PAIP1* | 2.0x10^-2^ | -0.39 | 2.6x10^-8^ | 92,634 |
| rs10941640 | *PAIP1* | 3.5x10^-2^ | 0.37 | 1.1x10^-5^ | 0 |
| rs6866325 | *PAIP1* | 1.2x10^-2^ | -0.39 | 2.0x10^-5^ | 0 |
| rs7708072 | *C5orf34* | 4.2x10^-2^ | 0.37 | 3.1x10^-5^ | 81,827 |
| chr5:43598333:I | *C5orf34* | 4.4x10^-2^ | 0.37 | 6.5x10^-5^ | 83,060 |
