# Supplementary material for: Mapping Variation in Cellular and Transcriptional Response to 1,25-Dihydroxyvitamin D3 in Peripheral Blood Mononuclear Cells
Source: PLoS One. 2016 Jul 25;11(7):e0159779. doi: 10.1371/journal.pone.0159779 (PMC4959717; doi:10.1371/journal.pone.0159779)
Supplement: S7 Table — Enriched pathways shown are at a p-value threshold of 0.05. The top 8 pathways were statistically significant at a FDR < 0.10. B-H p-value* = Benjamini-Hochberg multiple testing corrected p-value. (DOCX) [file pone.0159779.s013.docx]

**S7 Table. Gene set enrichment analysis of significantly differentially expressed (DE) genes (FDR < 0.01), using Ingenuity Pathway Analysis (IPA) software.** Enriched pathways shown are at a p-value threshold of 0.05. The top 8 pathways were statistically significant at a FDR < 0.10. **B-H p-value*** = Benjamini-Hochberg multiple testing corrected p-value.

| **Ingenuity Canonical Pathways** | **p-value** | **B-H p-value** | **Genes** |
| --- | --- | --- | --- |
| TREM1 Signaling | 4x10^-7^ | 2 x 10^-4^ | *TREM1,ICAM1,NLRP3,TLR8,CIITA,CCL3,TLR4,NOD2,PLCG2,TLR6,CASP1,CD86,IL1B,CCL7* |
| Granulocyte Adhesion and Diapedesis | 2x10^-5^ | 4x10^-3^ | *FPR3,ICAM1,C5AR1,FPR2,CCL22,CXCL5,MMP25,CCL3,CXCL6,FPR1,CLDN23,ITGAM,CCL8,CCL3L1,CCL3L3,IL1B,CXCL1,TNFRSF1B,CCL7* |
| T Helper Cell Differentiation | 6x10^-4^ | 6.5x10^-2^ | *ICOS,HLA-DMB,IL10RB,IL10RA,IFNGR2,CD86,IL2RA,TNFRSF1B,ICOSLG/LOC102723996* |
| Role of Pattern Recognition Receptors in Recognition of Bacteria and Viruses | 7x10^-4^ | 6.5x10^-2^ | *PTX3,NLRP3,C5AR1,TLR8,TLR4,NOD2,PRKCD,PLCG2,TLR6,CASP1,OSM,IL1B,RIPK2* |
| VDR/RXR Activation | 7x10^-4^ | 6.5x10^-2^ | *SERPINB1,CAMP,CYP24A1,GADD45A,PRKCD,CD14,NCOR2,CEBPB,THBD,RXRA* |
| Role of IL-17A in Arthritis | 1x10^-3^ | 7.4x10^-2^ | *CXCL1,MAPK13,CXCL5,PTGS2,RPS6KA1,MAPKAPK2,CXCL6,CCL7* |
| Role of IL-17A in Psoriasis | 1x10^-3^ | 7.4x10^-2^ | *S100A9,CXCL1,CXCL5,CXCL6* |
| Sulfate Activation for Sulfonation | 1x10^-3^ | 8.1x10^-2^ | *PAPSS1,PAPSS2* |
| Primary Immunodeficiency Signaling | 3x10^-3^ | 0.13 | *BTK,LCK,ICOS,CIITA,ADA,CD3D* |
| Role of IL-17F in Allergic Inflammatory Airway Diseases | 5x10^-3^ | 0.20 | *IL1B,CXCL1,CXCL5,RPS6KA1,CXCL6,CCL7* |
| LPS/IL-1 Mediated Inhibition of RXR Function | 7x10^-3^ | 0.23 | *CPT1A,CHST7,PAPSS2,CHST15,TLR4,LY96,CAT,IL1B,XPO1,CD14,PPARGC1B,HS3ST1,SLC27A3,RXRA,TNFRSF1B,ACSL1* |
| MIF-mediated Glucocorticoid Regulation | 7x10^-3^ | 0.23 | *TLR4,PLA2G4A,LY96,CD14,PTGS2* |
| LXR/RXR Activation | 7x10^-3^ | 0.23 | *TLR4,LYZ,LY96,CD36,CD14,IL1B,NCOR2,PTGS2,TNFRSF1B,RXRA,CCL7* |
| Agranulocyte Adhesion and Diapedesis | 7x10^-3^ | 0.23 | *ICAM1,C5AR1,CCL22,CXCL5,MMP25,CCL3,CXCL6,CLDN23,CCL8,CCL3L1,CCL3L3,IL1B,CXCL1,CCL7* |
| Uracil Degradation II (Reductive) | 9x10^-3^ | 0.23 | *DPYSL2,UPB1* |
| Thymine Degradation | 9x10^-3^ | 0.23 | *DPYSL2,UPB1* |
| Mitochondrial Dysfunction | 1.2x10^-2^ | 0.30 | *COX7B,ATP5G1,UCP2,CPT1A,CAT,COX5A,TRAK1,NDUFAF2,UQCRC1,CYB5R3,NDUFAB1,UQCRB* |
| CMP-N-acetylneuraminate Biosynthesis I (Eukaryotes) | 1.4x10^-2^ | 0.33 | *NAGK,CMAS* |
| Macropinocytosis Signaling | 1.6x10^-2^ | 0.35 | *PRKCD,PLCG2,HGF,USP6NL,CD14,ITGB8,PDGFB* |
| Type I Diabetes Mellitus Signaling | 1.6x10^-2^ | 0.35 | *HLA-DMB,IFNGR2,CD86,BID,IL1B,SOCS2,MAPK13,TNFRSF1B,CD3D* |
| MIF Regulation of Innate Immunity | 1.8x10^-2^ | 0.38 | *TLR4,PLA2G4A,LY96,CD14,PTGS2* |
| Purine Ribonucleosides Degradation to Ribose-1-phosphate | 2x10^-2^ | 0.40 | *ADA,PGM2* |
| Toll-like Receptor Signaling | 2.2x10^-2^ | 0.42 | *TLR4,LY96,TLR6,TLR8,CD14,IL1B,MAPK13* |
| Communication between Innate and Adaptive Immune Cells | 2.4x10^-2^ | 0.42 | *TLR4,CCL3L3,TLR6,TLR8,CD86,IL1B,CCL3* |
| iNOS Signaling | 2.5x10^-2^ | 0.42 | *TLR4,LY96,IFNGR2,CD14,MAPK13* |
| γ-linolenate Biosynthesis II (Animals) | 2.6x10^-2^ | 0.42 | *SLC27A3,CYB5R3,ACSL1* |
| Mitochondrial L-carnitine Shuttle Pathway | 2.6x10^-2^ | 0.42 | *CPT1A,SLC27A3,ACSL1* |
| Differential Regulation of Cytokine Production in Macrophages and T Helper Cells by IL-17A and IL-17F | 3x10^-2^ | 0.47 | *IL1B,CXCL1,CCL3* |
| IL-6 Signaling | 3.6x10^-2^ | 0.50 | *TNFAIP6,CYP19A1,CD14,IL1B,MAPK13,CEBPB,TNFRSF1B,MAPKAPK2,MCL1* |
| p53 Signaling | 3.6x10^-2^ | 0.50 | *RB1,GADD45B,GADD45A,THBS1,GNL3,HIF1A,DRAM1,PTEN* |
| iCOS-iCOSL Signaling in T Helper Cells | 3.6x10^-2^ | 0.50 | *LCK,ICOS,HLA-DMB,IL2RA,VAV1,CD3D,ICOSLG/LOC102723996,PTEN* |
| Cardiolipin Biosynthesis II | 3.9x10^-2^ | 0.52 | *PGS1* |
| Hepatic Cholestasis | 4.4x10^-2^ | 0.57 | *TLR4,LY96,CYP27A1,PRKCD,ADCY3,CD14,OSM,IL1B,TNFRSF1B,RXRA,ADCY7* |
| IL-17A Signaling in Fibroblasts | 4.5x10^-2^ | 0.57 | *MAPK13,CXCL5,CEBPB,CCL7* |
| Hepatic Fibrosis / Hepatic Stellate Cell Activation | 4.9x10^-2^ | 0.60 | *TLR4,LY96,ICAM1,HGF,IL10RA,IFNGR2,CD14,IL1B,ECE1,TNFRSF1B,PDGFB,COL7A1* |
