# Supplementary material for: Mapping Variation in Cellular and Transcriptional Response to 1,25-Dihydroxyvitamin D3 in Peripheral Blood Mononuclear Cells
Source: PLoS One. 2016 Jul 25;11(7):e0159779. doi: 10.1371/journal.pone.0159779 (PMC4959717; doi:10.1371/journal.pone.0159779)
Supplement: S8 Table — These response eQTLs are significant at a FDR < 0.10. The strength of the FAIRE-seq peaks is indicated by the Peak p-values and FDR, as well as the Peak fold enrichment values. (DOCX) [file pone.0159779.s014.docx]

**S8 Table. Response *cis*-eQTLs found in open chromatin regions detected by FAIRE-seq.** These response eQTLs are significant at a FDR < 0.10. The strength of the FAIRE-seq peaks is indicated by the Peak p-values and FDR, as well as the Peak fold enrichment values.

| **SNP** | **Gene** | **T-statistic** | **P-value** | **FDR** | **Beta** | **Peak P-value** | **Peak FDR** | **Peak fold enrichment** |
| --- | --- | --- | --- | --- | --- | --- | --- | --- |
| rs7520303 | *ETV3L* | 6.73 | 2.14x10^-9^ | 6.13x10^-5^ | 0.85 | 2.02x10^-11^ | 1.51x10^-19^ | 22.0 |
| rs12913835 | *EHD4* | 5.21 | 1.3 x10^-6^ | 2x10^-2^ | 0.72 | 2.2 x10^-5^ | 1.3x10^-5^ | 8.5 |
| rs6946706 | *ZNHIT1* | 4.94 | 4.07x10^-6^ | 4x10^-2^ | 0.87 | 1.76x10^-6^ | 3.5x10^-12^ | 14.3 |
