# Supplementary material for: Mapping Variation in Cellular and Transcriptional Response to 1,25-Dihydroxyvitamin D3 in Peripheral Blood Mononuclear Cells
Source: PLoS One. 2016 Jul 25;11(7):e0159779. doi: 10.1371/journal.pone.0159779 (PMC4959717; doi:10.1371/journal.pone.0159779)
Supplement: S9 Table — (DOCX) [file pone.0159779.s015.docx]

**S9 Table. Genes whose transcription responses are associated with I_max_ at a FDR<0.2.**

| **Gene** | **Beta** | **P-value** | **FDR** |
| --- | --- | --- | --- |
| *ZNF571* | -1.05 | 2.68 x 10^-5^ | 0.17 |
| *FNTA* | -1.16 | 4.26 x 10^-5^ | 0.17 |
| *GALNT4* | -0.82 | 9.74 x 10^-5^ | 0.17 |
| *PYCRL* | -1.48 | 1.04 x 10^-4^ | 0.17 |
| *HARBI1* | -1.62 | 1.16 x 10^-4^ | 0.17 |
| *UQCRC2* | 0.96 | 1.25 x 10^-4^ | 0.17 |
| *RASL11A* | 1.65 | 1.60 x 10^-4^ | 0.17 |
| *NOTCH3* | 1.45 | 1.60 x 10^-4^ | 0.17 |
| *ABCG1* | 2.01 | 1.65 x 10^-4^ | 0.17 |
| *PPP2R1A* | -1.11 | 1.90 x 10^-4^ | 0.17 |
| *GEMIN7* | -1.37 | 1.93 x 10^-4^ | 0.17 |
| *SMARCD3* | 1.55 | 2.18 x 10^-4^ | 0.17 |
| *SERPINA11* | -2.50 | 2.20 x 10^-4^ | 0.17 |
| *KNCN* | -1.33 | 2.61 x 10^-4^ | 0.19 |
| *PCSK6* | -1.57 | 2.88 x 10^-4^ | 0.19 |
| *PTGR2* | 1.20 | 2.97 x 10^-4^ | 0.19 |
