# Supplementary material for: Mapping Variation in Cellular and Transcriptional Response to 1,25-Dihydroxyvitamin D3 in Peripheral Blood Mononuclear Cells
Source: PLoS One. 2016 Jul 25;11(7):e0159779. doi: 10.1371/journal.pone.0159779 (PMC4959717; doi:10.1371/journal.pone.0159779)
Supplement: S10 Table — Genes associated with Imax were detected using a Bayesian approach implemented in the statistical program Sherlock [54]. The strength of the association between the genes and Imax is given by the overall log10 of Bayes factor (LBF). (DOCX) [file pone.0159779.s016.docx]

**S10 Table.** **Putative I_max_-associated genes.** Genes associated with Imax were detected using a Bayesian approach implemented in the statistical program Sherlock . The strength of the association between the genes and I_max_ is given by the overall log_10_ of Bayes factor (**LBF**).

| **Information on gene associated with I_max_** | | | | **Information on SNP associated with transcriptional response of corresponding gene, and with I_max_** | | | | |
| --- | --- | --- | --- | --- | --- | --- | --- | --- |
| **Gene** | **LBF** | **P-value** | **FDR** | **SNP** | **SNP Location** | **eQTL P-value** | **GWAS P-value** | **LBF of SNP** |
| *PAIP1* | 2.51 | 5.59x10^-6^ | 0.03 | rs6451692 | chr5: 43,433,735 | 2.01x10^-2^ | 2.55x10^-8^ | 2.51 |
| *ZNF649* | 2.32 | 5.59x10^-6^ | 0.03 | rs12459256 | chr19: 52,323,088 | 3.84x10^-4^ | 1.82x10^-5^ | 2.32 |
| *GORAB* | 1.06 | 8.94x10^-5^ | 0.27 | rs6427252 | chr1: 170,409,400 | 2.10x10^-4^ | 8.42x10^-4^ | 1.06 |
| *CAMK1G* | 0.85 | 1.90x10^-4^ | 0.43 | rs17014822 | chr1: 209,756,470 | 4.44x10^-4^ | 6.63x10^-4^ | 0.85 |
| *RAD18* | 0.79 | 2.46x10^-4^ | 0.44 | rs73132887 | chr3: 8,823,195 | 3.27x10^-4^ | 3.66x10^-4^ | 0.79 |
| *FGD2* | 0.69 | 3.80x10^-4^ | 0.57 | rs831504 | chr6: 36,988,364 | 9.28x10^-3^ | 1.04x10^-4^ | 0.69 |
| *LIN7A* | 0.60 | 5.59x10^-4^ | 0.61 | rs1163656 | chr12: 81,337,458 | 1.63x10^-3^ | 1.32x10^-4^ | 0.60 |
| *SMIM14* | 0.58 | 5.81x10^-4^ | 0.61 | rs11937734 | chr4: 39,482,848 | 5.70x10^-4^ | 2.41x10^-3^ | 0.58 |
| *TMEM8C* | 0.56 | 6.37x10^-4^ | 0.61 | rs3793627 | chr9: 136,407,659 | 3.14x10^-4^ | 1.69x10^-2^ | 0.56 |
| *B3GNT2* | 0.55 | 6.82x10^-4^ | 0.61 | rs2122382 | chr2: 62,326,484 | 7.03x10^-4^ | 1.18x10^-3^ | 0.55 |
| *ZNF385D* | 0.47 | 8.94x10^-4^ | 0.67 | rs6774929 | chr3: 21,480,184 | 1.11x10^-3^ | 4.66x10^-4^ | 0.47 |
| *ZFR* | 0.47 | 8.94x10^-4^ | 0.67 | rs11948227 | chr5: 32,273,114 | 9.96x10^-3^ | 1.70x10^-4^ | 0.47 |
