# Supplementary material for: Mapping Variation in Cellular and Transcriptional Response to 1,25-Dihydroxyvitamin D3 in Peripheral Blood Mononuclear Cells
Source: PLoS One. 2016 Jul 25;11(7):e0159779. doi: 10.1371/journal.pone.0159779 (PMC4959717; doi:10.1371/journal.pone.0159779)
Supplement: S11 Table — (DOCX) [file pone.0159779.s017.docx]

**S11 Table. List of loci highlighted in this study, and their respective phenotypic associations reported in the GWAS catalogue.**

| **Analysis** | **SNPs** | **Genes** | **GWAS catalogue reported traits** |
| --- | --- | --- | --- |
| Response eQTL mapping | rs74116976, rs7520303 | *ETV3L* | attention deficit hyperactivity disorder, conduct disorder |
|  | rs11070354,rs12913835 | *EHD4* | NA |
|  | rs7311057 | *PARPBP* | NA |
|  | rs59937851, rs6946706 | *ZNHIT1* | NA |
|  | rs7178702 | *SPESP1* | NA |
|  | rs10282056 | *COBL* | Wegener's granulomatosis, type I diabetes mellitus, post-traumatic stress disorder** |
|  | rs62014366 | *VWA9* | NA |
|  | rs7779605 | *CPED1* | bone mineral density |
|  | rs3848646 | *LRRC25* | NA |
|  | rs8081606 | *UNK* | NA |
|  | rs3783273 | *FRMD6* | hippocampal atrophy (Alzheimer's disease), brain volume measurement (Alzheimer's disease), protein C measurement, brain measurement, lung carcinoma |
|  | rs7698085 | *KIAA1211* | NA |
| Genes nearby top I_max_ GWAS SNP in Chr5 | rs6451692 | *PAIP1* | NA |
|  | rs6451692 | *ZNF131* | age at menarche, body mass index |
|  | rs6451692 | *NIM1K* | NA |
|  | rs6451692 | *C5orf34* | NA |
|  | rs6451692 | *C5orf28/TMEM267* | NA |
|  | rs6451692 | *NNT* | brain measurement |
|  | rs6451692 | *ANXA2R* | NA |
|  | rs6451692 | *CCL28* | hydroxy-leucine measurement (metabolite measures - Atherosclerosis Risk) |
| Top I_max_ SNP-Gene Associations (*cis*) – from Sherlock | rs6451692 | *PAIP1* | NA |
|  | rs12459256 | *ZNF649* | NA |
|  | rs6427252 | *GORAB* | waist-to-hip ratio adjusted for body mass index, atrial fibrillation, |
|  | rs17014822 | *CAMK1G* | obesity, amyotrophic lateral sclerosis, |
|  | rs73132887 | *RAD18* | NA |
|  | rs831504 | *FGD2* | acne |
|  | rs1163656 | *LIN7A* | age at onset, age-related cataract, dental caries, |
|  | rs11937734 | *SMIM14* | NA |
|  | rs3793627 | *TMEM8C* | NA |
|  | rs2122382 | *B3GNT2* | psoriasis, rheumatoid arthritis, ankylosing spondylitis |
|  | rs6774929 | *ZNF385D* | bipolar disorder, waist-hip ratio, partial epilepsy, periodontitis, airway wall thickness measurement, bipolar disorder, attention deficit hyperactivity disorder, |
|  | rs11948227 | *ZFR* | NA |
| Top I_max_ SNP-Gene Associations (*trans*) | rs1893662 | *PCSK6* | functional laterality (handedness in dyslexia), dyslexia |
|  | rs1893662 | *SMARCD3* | serum IgG glycosylation measurement |
|  | rs1893662 | *RASL11A* | electrocardiography and QT interval (electrocardiographic and heart rate variability traits), alcohol dependence |
|  | rs6451692 | *KNCN* | NA |
